# Research Progress for the Clinical Application of Circulating Tumor Cells in Prostate Cancer Diagnosis and Treatment

**DOI:** 10.1155/2021/6230826

**Published:** 2021-01-08

**Authors:** Mei Yang, Xiaotian Zhang, Lixia Guo, Xiumin Liu, Jing Wu, Hongquan Zhu

**Affiliations:** ^1^Department of Clinical Laboratory, The Second Hospital of Jilin University, Changchun, Jilin, China; ^2^Experimental Center, Medical College of Tibet University, Lhasa, Tibet Autonomous Region, China

## Abstract

Prostate cancer is a life-threatening and highly heterogeneous malignancy. In the past decade, circulating tumor cells (CTCs) have been suggested to play a critical role in the occurrence and progression of prostate cancer. In particular, as the “seed” of the cancer metastasis cascade, CTCs determine numerous biological behaviors, such as tumor invasion into adjacent tissues and migration to distant organs. Many studies have shown that CTCs are necessary in the processes of tumor progression, including tumorigenesis, invasion, metastasis, and colonization. Furthermore, CTCs express various biomarkers relevant to prostate cancer and thus can be applied clinically in noninvasive tests. Moreover, CTCs can serve as potential prognostic targets in prostate cancer due to their roles in regulating many processes associated with cancer metastasis. In this review, we discuss the isolation and detection of CTCs as predictive markers of prostate cancer, and we discuss their clinical application in the diagnosis and prognosis of prostate cancer and in monitoring the response to treatment and the prediction of metastasis.

## 1. Introduction

Prostate cancer (PCa) is one of the most common malignancies affecting men in the USA and one of the major causes of cancer-related death [1]. The incidence of PCa is 104.1 per 100,000 population in the US, according to data from 2012 to 2016 [2]. The estimated new cases of PCa account for 21%, ranking first among all male cancers in the US. As of 2020 [3], the incidence of PCa has also increased in China [4, 5]. Several factors have been linked to the risk of PCa, including age, family history, genetic susceptibility, race, and others [6]. Generally, the symptoms of early-stage PCa are not obvious, and by the time of diagnosis, the disease has progressed to an advanced stage with the possibility of distant metastasis [7]. Traditional diagnostic methods for PCa include histological examination of biopsied tissue, imaging via modalities such as magnetic resonance imaging (MRI), and measurement of serum tumor markers, such as prostate-specific antigen (PSA) [1, 8–10]. However, traditional imaging-based detection methods lead to cumulative radiation damage, while biopsy of such a small organ causes trauma and does not provide insight into the dynamic changes in the patient's condition. Additionally, serum tumor markers such as PSA have been shown to have low sensitivity and specificity [11–14]. Therefore, researchers seek a new method for prostate cancer detection based on circulating tumor cells (CTCs) [15, 16].

The concept of CTCs was first proposed in 1869 based on their appearance in the peripheral blood of patients with metastatic cancer, which was similar to that of primitive tumor cells [17]. CTC enrichment, detection, and downstream analysis are hampered by many obstacles due to two significant characteristics of CTCs: their rarity and heterogeneity [18, 19]. To survive and spread into distant organs, tumor cells must undergo a series of processes [20], which results in the destruction of most CTCs. Only a small proportion of CTCs ultimately survive, for an approximate frequency of 1 CTC per 10^6^-10^7^ blood cells [21]. In terms of heterogeneity, considerable genotypic and phenotypic diversity is observed among tumor cells in different tumor foci or subsets [22, 23], which further complicates the isolation and identification of CTCs. Therefore, the basis of all studies of CTCs is separation and enrichment. The sorting used to isolate CTCs mainly includes physical methods [24], such as separation techniques based on cell volume, deformability, density, and membrane characteristics, as well as immunological methods, such as the detection of epithelial cell adhesion molecule (EpCAM) [25], cytokeratin (CK) [26], and epidermal growth factor receptor (EGFR) [27] expression. The only CTC separation and counting system certified by the US FDA is the CELLSEARCH System, which employs immunomagnetic beads coated with EpCAM. Due to the heterogeneity among CTCs and the process of epithelial-mesenchymal transition (EMT) [28], the separation efficiency of the CELLSEARCH system is still not ideal [29]. In recent years, researchers have developed many novel isolation techniques aimed at providing a method that offers greater sensitivity, specificity, and accuracy for CTC enrichment. For example, Ko and his group developed a microchip platform that combines negative immunomagnetic selection and on-chip in situ RNA profiling [30]. Chen et al. reported a novel 3D-printed microfluidic device functionalized with anti-EpCAM antibodies [31], and other researchers developed a customized membrane platform based on surface-enhanced Raman spectroscopy (SERS) analysis [32] (Table 1).

**Figure 1:**
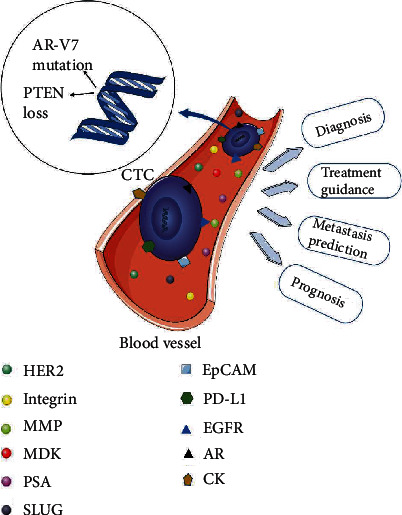
Biomarkers relevant to CTCs in prostate cancer progression. EpCAM, PD-L1, AR, and CK are expressed on CTCs in patients with prostate cancer; SLUG and integrin, MDK, MMP, PSA, EGFR, and HER2 are overexpressed in blood circulation; and MDK, MMP, PSA, EGFR, and HER2 may be expressed on CTCs during tumor metastasis and invasion. AR: androgen receptor; AR-V7: androgen receptor splice variant 7; CK: cytokeratin; EGFR: epidermal growth factor receptor; EpCAM: epithelial cell adhesion molecule; HER2: human epidermal growth factor receptor 2: MDK: midkine; MMP: matrix metalloproteases; PD-L1: programmed cell death protein-1 ligand; PSA: prostate-specific antigen; PTEN: phosphate and tensin homolog.

The clinical application of enriched CTCs is expected, and many studies have reported that CTCs serve as potential biomarkers in breast, lung, prostate, and pancreatic cancer. Rack et al. investigated the independent prognostic relevance of CTCs both before and after chemotherapy in a trial of breast cancer patients [33]. Su and his colleagues showed that monitoring the genomic changes of individual CTCs offers a method to evaluate tumorigenesis, the response to treatment, and the drug resistance of small-cell lung cancer (SCLC) [34]. Another study concluded that epithelial-mesenchymal hybrid CTCs (H-CTCs) may be a better indicator of metastasis, while epithelial-CTCs (E-CTCs) are a significant independent predictor of overall survival (OS) among pancreatic cancer patients [35]. In addition, many studies of CTCs in relation to PCa have also been reported, such as the study by El-Heliebi's group, which showed that CTCs of castration-resistant prostate cancer (CRPC) patients show positivity for androgen receptor splice variant 7 (AR-V7), androgen receptor full length (AR-FL), and PSA, which might contribute to the diagnosis or prognosis of PCa [36]. Another study showed that a high CTC count may contribute to the identification of high-risk PCa patients with occult metastases at the time of diagnosis [37]. Furthermore, the results of Hench's team indicated that mRNA analysis of CTCs in metastatic CRPC (mCRPC) is applied to monitor the responses to enzalutamide therapy and other treatments [38]. An increasing number of studies have suggested that CTCs may be a promising biomarker associated with PCa diagnosis, treatment, metastasis, and prognosis [39–42]. In this review, we aimed to discuss the clinical applications of CTCs in PCa to provide a reference for clinicians and researchers (as shown in Figure 1).

## 2. Diagnosis and Risk Assessment

CTCs are tumor cells that are shed from primary tumor lesions. After shedding, they enter the blood circulation spontaneously or passively [44, 45]. In addition to blood, they can also be detected in body fluids such as urine, hydrothorax, and ascites [46]. Recently, studies on CTCs have demonstrated a variety of molecular characteristics, including the expression of various surface markers, indicating that heterogeneous phenotypes are present and that these cells can be applied in methods beyond simple counting [44, 47, 48]. Therefore, the use of CTCs for the diagnosis and risk assessment of PCa has become a promising research area.

### 2.1. Diagnosis

To date, several studies have demonstrated the potential value of CTCs for PCa diagnosis. As reported in a study, scholars developed a new high-throughput capture chip for size-based CTC detection and separation on a microfluidic platform. Their chip achieved a capture rate of >95% for LNCAP-C4-2 PCa cells, and through optimization of the microstructure isolation rate and sorting limit, the seized cells were largely unharmed, suggesting that separated CTCs could still be analyzed for clinical diagnosis [49]. At the same time, researchers developed an optofluidic system using laser illumination for the isolation of modified CTCs from a combination of folic acid- (FA-) modified homologous red blood cells and tumor cells [50]. Their analyses showed a capture rate for CTCs exceeding 90% and a separation purity exceeding 92%, with the captured CTCs retaining membrane and functional integrity. Therefore, noninvasive and precise isolation methods for CTCs have shown great potential for use in the early diagnosis of PCa. In addition to improving separation devices, scientists have worked to improve methods for peripheral blood storage, to optimize cell conditions before processing, to increase the CTC capture rate, and to increase the viability of isolated cells. Wong et al. preserved peripheral blood by combining hypothermic preservation with countercooling-induced platelet activation and then used the microfluidic technology CTC-iChip to isolate CTCs [51]. Their results demonstrated an overall concordance of 92% for CTC-related genetic material between preserved and fresh blood and found that the captured CTCs retained intact RNA suitable for analysis by RNA-seq and single-cell RT-PCR. Therefore, the isolated cells could be used to detect the specific transcripts of patients with PCa, and their isolation technology could become the basis of a variety of diagnostic approaches. These findings improve the sorting rate by adopting various strategies so that CTCs can better assist in the diagnosis of PCa.

### 2.2. Risk Assessment

In addition to methods for diagnosing PCa, techniques are needed to evaluate the stage of PCa and the corresponding risk assessment for disease progression. There is a novel separation method for CTCs based on the specific binding of VAR2CSA malaria protein (rVAR2) and oncofetal chondroitin sulfate (ofCS) present on tumor cells before and after metastasis [52]. The results suggested that the sensitivity of this approach for the separation of CTCs was obviously improved compared with size-based capture, and by analyzing CTC counts for 25 PCa cases (stages I–IV), the conclusion showed that the CTC count correlated significantly with the cancer stage. Moreover, as shown by Drachenberg's group, the level of genomic instability in CTCs was detected utilizing quantitative three-dimensional (3D) telomere analysis [53]. They divided patients into three categories according to the risk for aggressive PCa, and their data indicated that the method may be a potential tool for assessing PCa patients' pretreatment risk. Some researchers have added CTCs as another biomarker to an index model including albumin, hemoglobin, lactate dehydrogenase (LDH), alkaline phosphatase (ALP), and PSA [54]. Their results proved that the addition of CTC count to the standard model provided more accurate risk assessment in terms of baseline and postbaseline prognosis of patients with mCRPC.

Referring to the above findings, for CTCs to be better used for the early diagnosis and risk assessment of prostate cancer, the key is to improve the sensitivity and specificity of CTC sorting. Multiple groups have established various new CTC sorting platforms, such as the Thermoresponsive NanoVelcro CTC purification system [55], a high-density microporous chip filter [56], in vivo cell collector technology [57], an immunomagnetic micro/nanoparticle system [58], and a high-throughput acoustic separation platform [59], to contribute to PCa diagnosis. However, the prognostic value of CTCs remains limited to preclinical research and is not currently able to be used clinically for patients. Large-scale prospective clinical trials are necessary to validate the potential value of CTC counts in both the diagnosis and risk monitoring of patients with PCa.

## 3. Treatment Guidance

Radical prostatectomy (RP), radiotherapy, and endocrine therapy are common strategies employed for PCa treatment [60, 61]. RP is mainly applied in cases of low- and medium-risk localized prostate cancer. Complications such as urinary incontinence are unfortunately common after surgery and seriously affect the quality of life of patients [62, 63]. Radiotherapy is more often used to treat medium- and high-risk localized PCa, and risk assessment of biochemical recurrence is still important when deciding to apply this treatment [64]. The survival and evolution of tumor cells depend on androgens, and almost all patients with PCa will progress to CRPC, the final stage of PCa progression, after androgen deprivation therapy (ADT) [65]. Pharmaceutical agents for the treatment of CRPC approved by drug regulatory authorities in China and around the world include AR-targeted medicines, such as abiraterone and enzalutamide, which block AR activation, and chemical agents, such as docetaxel and cabazitaxel, which inhibit the proliferation and induce the apoptosis of tumor cells [66, 67]. Certainly, the three main approaches to PCa therapy are not applied independently. Adjuvant radiotherapy is required with RP, and RP and radiotherapy must be combined with endocrine therapy [68]. At the same time, some studies published online have shown that AR splice variants are expressed on CTCs, and scholars have found that the presence of AR variants is related to resistance to AR-targeted therapies in PCa [69, 70]. Therefore, CTC-related assays have significant value for efficacy monitoring and resistance surveillance during therapy, and decisions or adjustments of treatment plans are likely to refer to CTC detection.

### 3.1. Efficacy Monitoring

Localized PCa is most commonly treated with RP or radiotherapy, and indicators relevant to CTCs may have reference value for treatment response screening. In a pilot analysis, researchers detected CTCs in 42% of localized prostate cancer patients before surgical tumor removal, and the CTC count showed a sharp decrease among 75% of these cases after surgical therapy [71]. Wark and colleagues compared CTC telomere results among high-risk cases of localized PCa (*n* = 100 cases) after 6 months of ADT, at 6 months after completion of RT, and at 36 months after initial treatment, and their results showed differences in treatment efficacy, indicating the potential value of CTCs in monitoring the efficacy of PCa treatment among patients [72]. Another study performed qRT-PCR to quantify RNA markers in CTCs related to PCa and showed that the detection of androgen receptor (AR) and programmed cell death protein-1 ligand (PD-L1) in CTCs could inform the decision of whether PCa patients should receive ADT or immunotherapy, respectively [73].

However, chemotherapy is recommended for advanced PCa or mCRPC. Studies have shown that taxane chemotherapy in patients with mCRPC might be a beneficial treatment after AR signaling inhibitor (ARSI) treatment failure, and AR-V7 status in CTCs reflects treatment efficacy [74]. Similar research reported that the effect of cabazitaxel seems to be independent of the AR-V7 status in CTCs; therefore, cabazitaxel may be a favorable option for patients with CTCs expressing AR-V7 [75]. Scher's group quantified digital pathological characteristics of CTCs in 179 mCRPC patients and found that high CTC phenotypic heterogeneity was related to better OS in patients treated with taxane, whereas low heterogeneity correlated with better OS in patients treated with ARSI [76]. Moreover, in a study on survival among mCRPC patients, researchers noted that AR-V7+ cases achieved a better OS when treated with taxane compared with ARSI, whereas AR-V7- cases achieved longer OS when treated with ARSI compared with taxane [77]. Taking the above data into consideration, the AR variant expression status of CTCs is able to predict the efficacies of different therapies for mCRPC and guide treatment decisions accordingly.

### 3.2. Resistance Surveillance

Endocrine therapy, along with immunological therapies, are promising therapeutic measures, but the emergence of drug resistance is a major problem impeding clinical application. Recent studies have shown that CTCs in peripheral blood may contribute to resistance evaluation in patients with PCa during treatment [78, 79]. Jan et al. characterized relevant RNA signatures to evaluate the PCS scores of CTCs in mCRPC after ARSI treatment, and their results demonstrated that ARSI-resistant (ARSI-R) cases had significantly higher PCS scores than ARSI-sensitive (ARSI-S) cases [55]. In addition, 8 cases that were ARSI-S initially later progressed to ARSI-R, and the PCS scores for these cases increased accordingly. Another study also indicated that one of the PCS subtypes similarly reflects enzalutamide resistance [80]. Some studies have also reported that midkine (MDK) is a chemokine that is upregulated in CRPC, and a research group found that elevated MDK expression on CTCs in metastatic hormone-sensitive prostate cancer (mHSPC) was significantly correlated with poor cancer-specific survival (CSS) [81]. They emphasized the potential role of MDK expression on CTCs for the study of resistance mechanisms. These novel noninvasive methods can facilitate early detection of medication resistance and allow for informed treatment selection.

## 4. Metastasis Prediction

**Figure 2:**
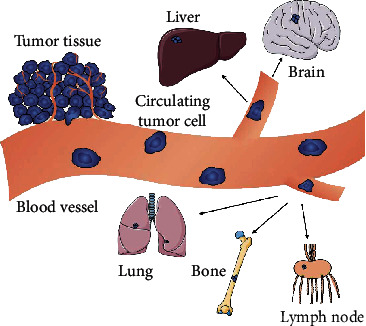
The metastatic cascade. After tumor formation, local tumor cells undergo EMT, invasive tumor cells invade blood vessels and migrate, and only a few of them metastasize to distant organs through immune escape mechanisms in the circulatory system. Then, they create a new microenvironment and build new metastases.

An estimated 90% of PCa-associated deaths are caused by metastasis [82]. CTCs play a critical role in the metastatic cascade, and CTCs circulate in peripheral blood after their release from primary tumors, extravasate, and form fatal metastases in different organs [83, 84] (shown in Figure 2). Minimal residual tumors that remain after primary treatment can lead to relapse and distant metastasis, and increasing evidence suggests that CTCs and bone marrow-derived disseminated tumor cells (BM-DTCs) provide biological insights into the dissemination and metastasis of PCa [85].

Cieslikowski' team concluded that a high CTC count might be considered a marker of metastatic high-risk PCa and could be used to identify PCa patients with occult metastases, with a sensitivity of 0.611, a specificity of 0.971, and an area under the curve of 0.901 [37]. Miyamoto and companions also demonstrated that an elevated high-risk digital CTC score before RP indicates microscopic spread into seminal vesicles and/or lymph nodes in a cohort study of localized PCa [69]. In another study, experimenters established a new classification system for CTCs with three PCS subtypes (PCS1-3), and their data showed that in patients with the PCS1 subtype, CTCs were more likely to disseminate and lead to progression to advanced disease compared with the other subgroups, even for low Gleason grade tumors [80]. In a multicenter randomized phase 3 trial of mCRPC treatment, researchers concluded that AR-V7 expression by CTCs was associated with various indicators of aggressive and advanced cancer, including high-volume bone disease, elevated PSA level, rapid progression, and short duration of efficacy for ADT [86]. Another group investigated changes in the expression profiles of 47 genes related to CRPC development in CTCs and identified 14 genes that were significantly differentially expressed in CTCs after CRPC relapse compared to before therapy, and the upregulated genes were related to steroidogenesis, AR signaling, and antiapoptosis pathways [81]. They summarized that changes in the expression levels of these 14 genes in CTCs might predict the recurrence of PCa.

Recent studies have also focused on the prediction of metastasis and the assessment of metastatic risk in PCa, and many of them have explored indicators relevant to how CTCs may regulate metastatic progression. Matrix metalloproteases (MMPs) are cell-secreted proteolytic enzymes associated with tumor invasion and metastasis, and the formation of new metastatic sites by CTCs involves MMP activity [87]. Dhar's team analyzed blood samples from mCRPC patients and found that 87% of CTCs secreted MMPs, that patients with PCa metastasis to bone or lymph nodes had higher CTC counts and greater MMP levels within CTCs, and that no CTCs and low levels of MMPs were detected in cases without metastasis [88]. These results demonstrated that a relative increase in MMP activity from CTCs implies that malignant processes are occurring, and the detection of such activity could contribute to assessments of PCa aggressiveness and metastasis as well as immune evasion by CTCs. Researchers observed that CTCs exhibit enhanced migration and EMT compared with cells derived from primary tumors by establishing a novel human xenograft CRPC mouse model [89]. Moreover, CTCs achieved stronger metastatic potential, in part through fibronectin regulation of integrin B1 and SLUG. From another study, it could be concluded that EGFR is expressed on tumor-initiating cells and is necessary for the formation of primary and secondary lesions by PCa cells [43]. Furthermore, EGFR expression was found on CTCs during PCa bone metastasis. Their data confirmed that EGFR promotes the survival of prostate CTCs that metastasized to bone and human epidermal growth factor receptor 2 (HER2) supports the growth of prostate tumor cells at metastatic sites.

In addition, an increasing number of scholars have paid attention to genomic analysis relevant to CTCs, which promises to be a foundation for the precise diagnosis and treatment of metastatic PCa [90]. A study performed whole-exome sequencing (WES) of 21 CTCs derived from aggressive PCa, and scientists identified more than 202,000 single-nucleotide variants (SNVs) and over 137,000 insertion-deletions (indels) [91]. In a relevant study, genomic analysis of chromosomal copy number alterations (CNAs) was carried out in CTCs, and the results indicated that genomic instability in CTCs is a sign of aggressive PCa, including increased AR expression, loss of BRCA2, and amplifications in chromosomal regions of PTK2, MYC, and NCOA2 [92]. Therefore, CTCs can be used to predict and assess the risk and progression of PCa dissemination through both counting and quantification of gene expression. Further research will determine how CTCs regulate tumor migration and growth via the characterization of specific proteins expressed by these cells in combination with other factors in PCa metastasis.

## 5. Prognosis

Studies have suggested that the CTC count, CTC phenotype heterogeneity, and expression of prostate-derived transcripts in CTCs may be useful for determining the prognosis of PCa [93–95]. A study reported that the CTC count is independently associated with progression-free survival (PFS) and OS in mCRPC patients treated with cabazitaxel [96]. Similarly, Danila and coworkers showed that a postchemotherapy CTC count of <5 cells/7.5 ml peripheral blood predicts longer survival in CRPC [97]. In 208 mCRPC patients from the MAINSAIL trial, researchers demonstrated that the CTC count of ≥5 cells/7.5 ml peripheral blood before treatment with docetaxel was significantly correlated with worse OS [98]. Additionally, an increase in the CTC count from <5 cells/7.5 ml to ≥5 cells/7.5 ml peripheral blood after three treatment cycles was associated with a significantly lower OS, whereas a decrease in the CTC count from ≥5 cells/7.5 ml to <5 cells/7.5 ml peripheral blood indicated a favorable prognosis.

PCa is a biologically heterogeneous disease, and various molecular alterations occur during tumorigenesis and progression [99–101]. Therefore, the detection of CTC phenotype heterogeneity and prostate-derived transcript expression in CTCs is likely of great value in determining prognosis. One study analyzed gene expression patterns in isolated CTC and CTC clusters from PCa patients by RT-PCR and found that high expression of stemness genes, which reflects an undifferentiated CTC phenotype, was associated with poor prognosis [102]. In a prospective study of abiraterone treatment, experimenters performed digital quantitation on prostate-derived transcript expression in CTCs, and their results suggested that an elevated digital CTC score pretreatment could predict poor OS and shorter radiographic PFS [69].

Another subject group evaluated AR-V7 expression on CTCs using a novel digital droplet PCR (ddPCR) assay, and they found that AR-V7 expression on CTCs correlates with prognosis following taxane chemotherapy and PFS in mCRPC [103]. In a cohort of 193 patients with progressive mCRPC, it was found that patients whose CTCs tested positive for nuclear-localized AR-V7 tended to have superior survival if treated with taxane chemotherapy [104]. Other researchers also reported that loss of tumor suppressor phosphate and tensin homolog (PTEN) gene expression occurs frequently in CRPC and promotes progression via the PI3K/AKT pathway [105, 106]. Punnoose's team assessed PTEN gene expression status in CTCs by fluorescence in situ hybridization (FISH) and reported that PTEN deletion in CTCs was associated with poor survival among mCRPC patients [107]. Together, these studies indicate that an increase in the CTC count, an undifferentiated CTC phenotype, AR-V7 expression by CTCs, and PTEN loss in CTCs may indicate a poor prognosis of PCa. The main references and their research results are listed in Table 2.

## 6. Summary

In this review, we discussed research progress related to the clinical application of CTCs, especially for the diagnosis of PCa and the evaluation of response to treatment and risk of metastasis. In summary, CTCs are involved in multiple processes of cancer progression and have been considered the “seed” of tumor dissemination. Accumulating evidence demonstrates that CTCs participate in various tumor processes, including tumorigenesis, invasion, migration, and colonization. In clinical practice, MRI and histological biopsy are common methods for PCa diagnosis, but these traditional methods have limitations. Since the identification of CTCs, multiple studies have demonstrated a potential role for these cells in the management of PCa, specifically for monitoring treatment efficacy and predicting metastasis and recurrence. However, the use of CTCs as a diagnostic marker in PCa remains controversial in clinical research.

Chemotherapy has long been the main treatment for advanced PCa. Classical chemotherapies for PCa include AR-targeted drugs that block the activation of AR and taxane chemicals that inhibit tumor cell proliferation or induce tumor cell apoptosis. Treatment guidance for PCa through CTC counting and analysis of surface protein and mutant gene transcript expression may be possible, but further research should also take into consideration more indicators, such as exosomes, circulating tumor DNA [108], long noncoding RNAs, microRNAs, and circulating RNAs, as potential biomarkers [109, 110]. A comprehensive approach is likely needed to more accurately determine the treatment efficacy, drug resistance, and prognosis of PCa patients during treatment.

In addition, CTCs can be applied to assess the metastasis risk in PCa through CTC counting and analysis of the expression of proteins through which these cells regulate the processes of metastasis. Based on studies of CTC biology, we need to integrate oncology and immunology approaches closely to explore the importance of CTCs in the mechanisms of metastasis and drug resistance. Such research will provide further insight into the potential value of CTCs for precise PCa diagnosis and treatment guidance.

## Figures and Tables

**Table 1:** The rationale and capture efficiency of each isolation platform. EpCAM: epithelial cell adhesion molecule; CK: cytokeratin; CTCs: circulating tumor cells; CRPC: castration-resistant prostate cancer; OS: overall survival; DPBS: Dulbecco phosphate-buffered saline; EGFR: epidermal growth factor receptor; PDAC: pancreatic ductal adenocarcinoma; SERS: surface-enhanced Raman spectroscopy.

Isolation platform	Rationale	Samples	Results	References
CELLSEARCH System (Veridex LLC)	Based on immunoaffinity (EpCAM and CK)	Blood samples with CRPC	CTC class showed a strong association with OS	Coumans et al. (2010) [25]

Lateral filter array microfluidic (LFAM) device	Based on size and immunoaffinity (EpCAM)	L3.6pl cells spiked in DPBS	Capture efficiency (98.7 ± 1.2%)	Chen et al. (2019) [26]
		MCF7 cells spiked in DPBS	Capture efficiency (93.8 ± 1.5%)	
		L3.6pl cells spiked in healthy blood	Capture efficiency (95.4 ± 1.1%)	
		Blood samples with metastatic colorectal cancer	Capture efficiency 100% (3.2 CTC/ml blood)	

Microfluidic graphene oxide chip	Based on immunoaffinity (EGFR, EpCAM, CK7/8, CD45)	Blood samples with metastatic prostate cancer	Capture efficiency 100%	Day et al. (2017) [43]

CaTCh FISH chip	Based on immunomagnetic and RNA profiling	Blood samples with PDAC	Capture efficiency 85.7%	Ko et al. (2017) [30]

3D-printed microfluidic devices	Based on size and immunoaffinity (EpCAM)	Cell lines (MCF-7, SW480, PC3, or 293T) spiked in PBS	Capture efficiency >90%	Chen et al. (2019) [31]

SERS platform	Based on size and SERS	HeLa cells and PC3 cells spiked in healthy blood	The spectrum showed obvious differences in different cells	Kaminska et al. (2019) [32]

**Table 2:** Main references and their research results in this review.

Clinical application	Clinical significance on prostate cancer	Reference
Diagnosis	Showed great potential for early diagnosis	Ren et al. [49], Hu et al. [50]
	The isolated CTC could be used to detect the transcripts and become the basis of diagnostic approaches	Wong et al. [51]

Risk assessment	CTC count correlated significantly with the cancer stage	Agerbæk et al. [52]
	The level of genomic instability in CTC may be a potential tool for assessing PCa patients' pretreatment risk	Drachenberg et al. [53]
	CTC count could help to provide more accurate risk assessment	Heller et al. [54]

Efficacy monitoring	CTC count showed a sharp decrease among 75% patients with localized prostate cancer after surgical therapy	Stott et al. [71]
	CTC telomere under different efficacies showed a clear difference	Wark et al. [72]
	Detection of AR and PD-L1 on CTC could help in the treatment options of PCa	Yin et al. [73]
	The AR-V7 expression status of CTC is able to predict the therapeutic efficacy for mCRPC	Onstenk et al. [75], Scher et al. [76], Thoma [77]

Resistance surveillance	The characterization of CTC can reflect the sensitivity of endocrine therapy	Jan et al. [55], You et al. [80], Josefsson et al. [81]

Metastasis/recurrence prediction	CTC count/CTC PCS score might be a marker of metastasis	Cieslikowski et al. [37], You et al. [80], Miyamoto et al. [69]
	The expression of AR-V7 on CTC was associated with the cancer aggressiveness	Taplin et al. [86]
	The expression levels of 14 genes in CTC may predict the recurrence of PCa	Josefsson et al. [81]
	CTC achieved stronger metastatic potential	Day et al. [43], Huaman et al. [89]

Prognosis	CTC count is associated with PFS and OS in PCa patients	de Kruijff et al. [96], Danila et al. [97], Vogelzang et al. [98]
	The expression of the phenotype and transcript on CTC has great value in determining prognosis	Miyamoto et al. [69], Kozminsky et al. [102], Tagawa et al. [103], Graf et al. [104], Punnoose et al. [107]

CTC: circulating tumor cell; PCa: prostate cancer; AR: androgen receptor; PD-L1: programmed cell death protein-1 ligand; AR-V7: androgen receptor splice variant 7; mCRPC: metastatic castration-resistant prostate cancer; PCS: prostate cancer classification system.

## Data Availability

All data generated or analyzed during this study are included in this article.
